# Characteristics of falls in mild and moderate Alzheimer’s disease

**DOI:** 10.1590/S1980-57642009DN30400013

**Published:** 2009

**Authors:** Eliane Mayumi Kato-Narita, Marcia Radanovic

**Affiliations:** 1 Physiotherapist, MsC, Behavioral and Cognitive Neurology Unit, Department of Neurology, University of São Paulo School of Medicine, São Paulo SP, Brazil.; 2 Neurologist, MD, PhD, Behavioral and Cognitive Neurology Unit, Department of Neurology, University of São Paulo School of Medicine, São Paulo SP, Brazil.

**Keywords:** falls, elderly, Alzheimer’s disease

## Abstract

The occurrence of falls in AD patients is frequent, and their consequences are cognitive decline, fractures and loss of independence. Many studies have addressed fall risk factors in order to establish effective prevention strategies.

**Objectives:**

We describe the frequency and characteristics of falls in a sample of AD patients and their main risk factors.

**Methods:**

We evaluated 40 subjects without cognitive impairment, and 45 AD patients, graded as CDR 1 and CDR 2.

**Results:**

Environmental hazard risks were the most frequent cause associated with falls in CDR 1 (41.4%) and CDR 2 (46.7%). Instability (31%) and dizziness (17.2%) were frequent causes of falls in the CDR 1 group, and this group showed the highest rate of recurrence (28%). In both groups of AD patients, indoors falls predominated (70.3 and 80% respectively for CDR 1 and 2). In our sample, the remaining factors studied were not associated with increasing risk for falls.

**Conclusions:**

These results reinforce the hypothesis that falls in AD are mutifactorial and that their risk factors are highly interconnected. Preventative strategies considering all aspects should be implemented most crucially eliminating environmental risks, maintaining constant presence of caregivers, and providing physical and functional stimulation, both in mild and moderate AD.

## Abstract (pt)

**Fundamentos:**

A ocorrência de quedas entre idosos com a doença de Alzheimer (DA) é muito frequente, e traz consequências graves como a piora cognitiva, fraturas e perda da independência. Muitos estudos foram realizados com o objetivo de identificar os fatores de risco associados às quedas, com o fim de propor estratégias de prevenção.

**Objetivos:**

O presente estudo buscou descrever a frequência e características das quedas em uma amostra de pacientes com DA, e seus principais fatores de risco.

**Métodos:**

Foram analisados 40 idosos sem alterações cognitivas e 45 com DA, classificados em CDR 1 e CDR 2.

**Resultados:**

Riscos ambientais foram associados às quedas tanto no grupo CDR 1 (41.4%) como CDR 2 (46.7%). Instabilidade (31%) e tontura (17.2%) foram frequentes no grupo CDR 1, sendo este o grupo que apresentou o maior índice de recorrência (28%). Em ambos os grupos com DA, as quedas ocorreram mais frequentemente dentro de casa (70,30 e 80% respectivamente para os grupos CDR 1 e 2).

**Conclusões:**

Estes resultados reforçam a hipótese de que as quedas em pacientes com DA são multifatoriais e seus fatores de risco altamente interconectados. Medidas preventivas devem ser realizadas, principalmente quanto a retirada de riscos ambientais, a presença constante de cuidador, e a estimulação física e funcional do paciente, tanto na fase leve como na moderada da DA.

## Introduction

Falls in the elderly are one of the most frequent causes of incapacity and death.^1^ Many studies have addressed the causes and risk factors for falls in community-dwelling elderly. One such risk factor is the existence of cognitive impairment.^2-5^

Elderly with dementia have a doubled to threefold risk for the occurrence of falls,^6^ probably due to motor impairment,^7^ attentional deficits,^8^ use of psychotropic medication and behavioral symptoms.^9-11^ Other risk factors include necessity of a walking-aid, functional impairment, history of recurrent falls, living in long-term residence, and presence of symptomatic orthostatic hypotension.^12-14^

Considering the consequences of falls in dementia (such as functional dependency, progression of the disease and death) and the high social cost and burden of caregivers, many studies have attempted to identify the risk factors for falls and to develop fall prevention strategies.^15,16^ However, studies on risk factors fail to consider the stage of dementia and most have been conducted in dementia care centers or hospitals, and therefore did not address patients who were living in their own homes. These reasons might have led to negative results in prevention strategies.

The objective of this study was to describe the frequency and characteristics of falls and their main risk factors in a sample of Alzheimer’s disease (AD) elderly, according to severity of the disease (CDR 1 and CDR 2).

## Methods

### Participants

All participants were 60 years and older and of both genders. Forty five AD patients were recruited from an outpatient service at a university hospital. The diagnosis of AD was based on NINCDS-ADRDA criteria.^17^ The Clinical Dementia Rating (CDR) was used to stage the severity of AD, classifying the disease into mild and moderate AD (CDR 1 and 2).^18^

The control group included forty elderly without cognitive impairment, recruited from the community and assessed by two neurologists, based on the criteria of the Mayo Older Normative Studies.^19^

For both groups, the exclusion criteria were: presence of vertigo or dizziness, vestibulopathy, episodes of loss of consciousness, other neurological diseases (stroke, Parkinson’s disease or parkinsonism, other causes of dementia), untreated depression, uncorrected visual impairment, severe hypoacusia and musculoskeletal alterations with limiting pain.

The study was approved by the local Ethics Committee (CAPPesq 311/04). All participants and/or their legal representatives signed an informed consent term prior to enrollment on the study.

### Procedures

All participants answered a questionnaire containing social-demographic data, and questions concerning the use of medications, walking-aids, as well as history of falls in the last twelve months along with their characteristics. Falls were described to caregivers as any event in which the elderly unintentionally came to rest on the floor or on a lower level to which he or she was standing, regardless of whether or not an injury was sustained. The MMSE was applied to patients and controls as the initial part of the cognitive assessment.

Controls and AD caregivers answered the functional questionnaire Disability Assessment for Dementia (DAD)^20^ and the Cornell depression scale^21^ in order to establish the functional status and the presence of depressive symptoms in both groups (patients and controls).

### Data analysis

Descriptive statistical analyses (mean, standard deviation, minimum and maximum values) were performed for demographic data and variables of interest (MMSE, duration of disease, Cornell, DAD and number of falls). Comparison of means among the three groups (controls, CDR 1 and CDR 2) was performed using one-way ANOVA with Tukey’s post-test. Student’s t test was employed for independent samples to compare continuous variables between two groups (CDR 1 and CDR 2). The distribution of frequencies for categorical variables (marital status, gender, use of medication, occurrence and recurrence of falls) was analyzed through the Chi-square test.

The sample was then subdivided into “fallers” and “non fallers” groups (maintaining the CDR classification within the two groups), and logistic regression analysis was performed in order to identify the risk variables for the occurrence of falls. The level of significance adopted for all analyses was 0.05.

## Results

### Characteristics of the participants

Table 1 shows the clinical and socio-demographic characteristics of the sample. Differences were observed among the three groups on the MMSE and DAD scores. CDR 2 patients differed from controls on the Cornell scores. There was a higher prevalence of single subjects in the control group. Table 2 shows the use of medications in each group.

**Table 1:** Clinical and socio-demographic characteristics of the control and AD groups.

Variable	Controls (n=40)	CDR 1 (n=25)	CDR 2 (n=20)	p (two–tailed)	Multiple comparison
Age (years)	74.5 (7.3)	77 (7)	77.6 (5.5)	0.1810	–
Schooling (years)	6.6 (48)	5.4 (4.9)	5.3 (4.6)	0.5070	–
Gender*					
M	18	8	4		
F	22	17	16	0.1480	
Marital status*					
Single	10	0	1		
Married	13	12	11	0.0060	
Widowed	12	13	8		
Divorced	5	0	0		
Duration of disease (mo)**	NA	34 (25.8)	50.8 (30.6)	0.0530	
Primitive reflexes*					
Yes	NA	15	12	0.0940	
No	NA	10	8		
MMSE	26.8 (3)	18.6 (3.9)	13.9 (5.3)	< 0.0001	All groups differ (p<0.0001)
Cornell**	NA	2.9 (2.7)	3.4 (2.3)	0.5670	–
DAD	100 (0)	80.7 (13.6)	67.5 (14.9)	< 0.0001	All groups differ (p<0.0001)

One-way ANOVA with Tukey's post-test;

* Chi-square test;

** Student's t test; NA, not applicable

**Table 2:** Number of subjects taking medications in the control and AD groups

Medication	Controls	CDR 1	CDR 2	p (two-tailed)
Anticholinesterasics	0	24	20	< 0.0001
Antidepressants	3	9	6	0.0160
Antipsychotics	0	6	6	0.0030
Muscular relaxing drugs	5	0	0	0.0500
Benzodiazepines	1	1	0	0.6760
Antihypertensives	19	9	12	0.5710
Antiarritmics	3	0	0	0.1730

Chi-square test.

### Characteristics of falls

In our study, 45% of the controls had fallen at least once within the preceding twelve months whilst in the AD group the frequencies were 56% and 55% for CDR 1 and CDR 2, respectively. Concerning the control group, the number of falls was 0.65/person, and five persons fell more than once. For the CDR 1 group, the number of falls was 1.16/person, and for CDR 2, 0.75; no significant difference between these groups was found. Considering only those who fell (“fallers”), the number of falls was 1.44, 2 and 1.36/person, respectively. Recurrence of falls did not differ between the groups (Table 3).

**Table 3:** Frequency and number of falls occurred in the last twelve months.

Variables	Controls	CDR 1	CDR 2	p (two-tailed)
Number of falls M (SD) – total group	0.65 (0.9)	1.16 (1.5)	0.75 (0.8)	0.190
Number of falls M (SD) – "fallers" group	1.44 (0.8)	2 (1.5)	1.36 (0.6)	
Occurrence of falls*				0.622
Yes	18 (45%)	14 (56%)	11 (55%)	
No	22 (55%)	11 (44%)	9 (45%)	
Recurrence of falls*				0.263
Yes	5 (12.5%)	7 (28%)	3 (15%)	
No	35 (87.5%)	18 (72%)	17 (85%)	

Student's t test

* Chi-square test.

The majority of falls in the CDR 1 group occurred indoors (mostly within the patient’s home – 79.3%), including the yard, and only 20.7% occurred outdoors (in streets or at unfamiliar places). Similar results were found in the CDR 2 group with frequencies of 80% and 20%, respectively. The occurrence of indoor falls was statistically more frequent among AD patients, regardless of disease severity (Table 4).

**Table 4:** Frequency of falls according to place of the occurrence.

Local	Indoors	Outdoors	p (two-tailed)
Controls	9 (50%)	9 (50%)	0.7389
CDR 1	23 (70.3%)	6 (30.7%)	0.0054
CDR 2	12 (80%)	3 (20%)	0.0035

Chi-square test

Environmental hazard risks were the most common cause of falls in our sample, both in CDR 1 and CDR 2 groups, being responsible for 41.4% and 46.7% of falls, respectively. Dizziness and instability were more frequently reported as causing falls in the CDR 1 group. For CDR 2 patients, 46.7% of reported falls had their cause classified as “ignored” or “not remembered”. (Table 5)

**Table 5:** Causes related to falls in AD patients.

Cause of fall N (%)	CDR 1	CDR 2	total AD group	p
Environmental hazard	12 (41.4%)	7 (46.7%)	19 (43.1%)	0.9157
Dizziness	5 (17.2%)	0 (0%)	5 (11.3%)	0.4109
Instability	9 (31%)	1 (6.6%)	10 (22.6%)	0.1464
Ignored	3 (10.4%)	7 (46.7%)	10 (22.6%)	0.0192

Chi-square test

### Risk factors for falls

In our sample, logistic regression analysis found no association between the variables age, duration of disease, number and type of medications used, presence of primitive reflexes, score on the DAD, score on Cornell or number or frequency of falls (raw data not shown).

## Discussion

Previous studies on falls in the elderly have confirmed the most relevant risk factors as being older age, history of previous falls, necessity of walking aid, presence of visual impairment, high number of drugs (mainly psychotropic drugs), balance disorders and functional impairment.^9,14,15,22-26^

Some of these factors have also been identified in studies with dementia, which have indicated other concurrent risk factors such as high grade periventricular white matter lesions, parkinsonism, peripheral neuropathy, diagnosis of dementia with Lewy body and vascular dementia.^7,27-32^ However, most of these studies did not consider the influence of some variables on the occurrence of falls, such as the etiology of the dementia, study setting (home, hospital or long-term care residences) or the stage of the disease, thus introducing some bias into the results.

This study included only AD patients, a factor that might explain why no powerful single predictor for the occurrence of falls was identified. Studies including various etiologies of dementia may introduce additional variables related to the specificities of each disease, thus making it hard to correctly estimate the impact of a given risk factor on the occurrence of falls.

In Brazil, most elders live in their own residences^33,34^ and not in residential care facilities or long-term care institutions, where many studies on falls have been conducted. It is well known that falls occur more frequently in these places because of lack of supervision, use of more psychotropic drugs to control behavioral disturbances and wandering, besides greater hazard risks^35,36^.

Depending on the severity of the dementia, motor signs are present and can include gait disturbances, extra-pyramidal motor impairment, rigidity and postural instability, all being strongly associated with falls. Moreover, behavioral disturbances such as attentional deficits, wandering and aggressiveness appear in different stages of dementia, contributing to the occurrence of falls.^37,38^ This underscores the importance of analyzing the occurrence of falls and their risk factors according to the stage of dementia.

We found a higher incidence of falls in our non-demented subjects than that described in a previous report in Brazill,^4^ registering approximately 30%. Several factors reported by our subjects could contribute to this higher incidence of falls, such as the use of public transportation (especially buses), having pets indoors, and climbing chairs to reach objects located in elevated places (such as cabinets).

The frequency of falls in mild and moderate AD (CDR 1 and 2) in our study was similar to that found in other studies, as was the rate of falls per patient.^5,6,12-14^

Eriksson et al. ^14^ found that elderly subjects with dementia that use more than four drugs have an increased risk for falls. The number of drugs are reported as a predictor of morbidity and mortality, and probably reflects the poor functional level of the elderly.^3^ Many studies^6,10,11,14,30,38,39^ have described the correlation between use of psychotropic and antidepressants drugs and increased risk of falls, as a result of drowsiness and motor deficits. However, this association was not found in our study.

In our study, almost 80% of the falls occurred indoors, a result similar to that reported by Soriano et al (77%).^40^ Mild AD patients remain independent and have less motor impairment, but frequently are not allowed to leave their homes unaccompanied, thus being more exposed to environmental hazard risks during their activities of daily life indoors. Additionally, they may have attentional deficits and poor risk judgment, particularly during gait, as cited by Yamaguchi.^41^

On the other hand, moderate AD patients have more motor impairments and greater spatial disorientation, demanding the constant presence of a caregiver rendering them even more limited, which might prevent the occurrence of falls outdoors. These patients become more dependent regarding walking and performing functional activities. The fear of falling leads to limitations in mobility in 20% to 55% of elderly subjects, and can cause reduction in muscular strength, agility and balance, and loss of independence,^40^ thus further increasing the risk for falls especially if the patient walks unattended.

Loss of functional capacity has been cited as either a cause or a consequence of falls, because of restrictions in mobility and consequent loss of strength and incapacity to perform activities.^42,43^ Although a decline in functional capacity according to DAD scores have been observed in both stages of AD, we found no association between functional disability and the occurrence of falls.

In our study, 43.2% of falls in the AD group were caused by environmental hazard risks, which caused slips or slides. Analyzing each group, 41.4% of falls in CDR 1, and 46.7% in CDR 2 patients were caused by factors such as stairs, wet floor, obstacles and using buses. Soriano et al. ^40^ found that almost 30% of falls were caused by the environment.

The high frequency of this particular risk factor shows that lack of attention might be an important component predisposing individuals to falls, both in mild and moderate AD. These findings show that an important intervention to prevent falls entails keeping the environment safe, in both stages of AD. However, changes in patient’s homes should not be so extensive so as to completely modify the characteristics that are familiar to the dweller and which serve as references to the patient, because these can potentially lead to spatial disorientation and confusion.

In the CDR 2 group, 46.7% of falls were of “ignored” cause, probably because the cognitive impairment prevents recall, and because most falls occurred in the absence of a witness caregiver. This might suggest that many falls occurred when the patient was alone, during walking or risky activities, and was unable to avoid them as a result of postural instability or lack of balance strategies. Dizziness and instability were frequent causes of fall in the CDR 1 group, which might explain the relatively higher frequency of recurrent falls in this group.

The actual frequency of occurrence of falls is difficult to estimate because of the following reasons: some falls are not remembered or go unseen by caregivers, some are not considered a fall according to the caregivers’ judgment and caregivers may underreport a fall if it occurred as a result of their own negligence. In a study conducted by Eriksson et al.,^14^ 24 falls (9%) were not reported by caregivers.

Our results suggest that more specific risk factors should be assessed, such as those addressed by Pelfolk et al.^6^ These researchers found that 36.1% of the variance in falls could be explained by being dependent in hygiene, displaying verbally disruptive/attention-seeking behavior, being able to rise from a chair, walking with assistive devices, and participating in outdoor walks, observed through logistic regression.

As falls are unpredictable events it is a laborious task to determine each underlying risk factor and interaction among them. Soriano et al.^40^ described a total of 25 risk factors for falls. This accounts for the fact that prevention strategies are hard to implement, and justifies why many preventive strategies are not as effective as desired.

These results reinforce the hypothesis that falls in AD are mutifactorial and that their risk factors are highly interconnected. Preventative strategies considering all aspects should be implemented, most crucially those eliminating environmental risks, maintaining constant presence of caregivers, and providing physical and functional stimulation, both in mild and moderate AD. Concluding, in our sample, previously well-established fall risks were not associated with an increase in the occurrence of falls in AD, in either mild or moderate stages. As cited by Eriksson et al.,^14^ “Conventional risk factors for falling explain a much smaller portion of the variations in falls among people with dementia than without dementia”, a point on which we agree.
